# Epidermoid cyst of the craniovertebral junction—A case report

**DOI:** 10.1002/ccr3.8726

**Published:** 2024-03-27

**Authors:** Duong Trung Kien, Nguyen Manh Hung, Nguyen Viet Duc, Duong Dinh Tuan, Luong Minh Quang, Pham Van Duong, Tran Viet Hoang, Vu Ngoc Anh

**Affiliations:** ^1^ Department of Neurosurgery Saint Paul Hospital Hanoi Vietnam; ^2^ Department of Surgery Hanoi Medical University Hanoi Vietnam

**Keywords:** cerebellopontine angle, craniovertebral junction, epidermoid cyst, microsurgery

## Abstract

An epidermoid cyst is a benign tumor in many locations. The symptoms of an epidermoid cyst depend on its location. The brain or spine MRI can confirm the lesion. Removing total decompression is the first choice in treatment with a symptomatic cyst.

## 1 INTRODUCTION

Intracranial epidermoid cyst accounts for approximately 1% of all intracranial tumors^1^, ^2^ with similar incidence in both sexes. The first symptoms most often occur in the fourth decade of life. It is a benign and slow‐growing lesion. The patients' symptoms depend on the location of the epidermoid cyst. MRI is more effective than CT because the sequences of MRI allow differentiating the epidermoid from the cerebrospinal fluid and non‐specific central nervous system lesions.^3^ Complete cyst excision with preservation of adjacent neuronal and vascular structures is essential to decrease post‐operative morbidity and recurrence.

## 2 CASE HISTORY/EXAMINATION

A 41‐year‐old female with progressive symptoms, including headache, vomiting, and balance disturbances that had been evolving about 10 months. She had no significant past medical history. Neurological examination presented intracranial hypertension and disorders in alternating movement.

## 3 METHOD

### 3.1 Radiological evaluation

**FIGURE 1:**
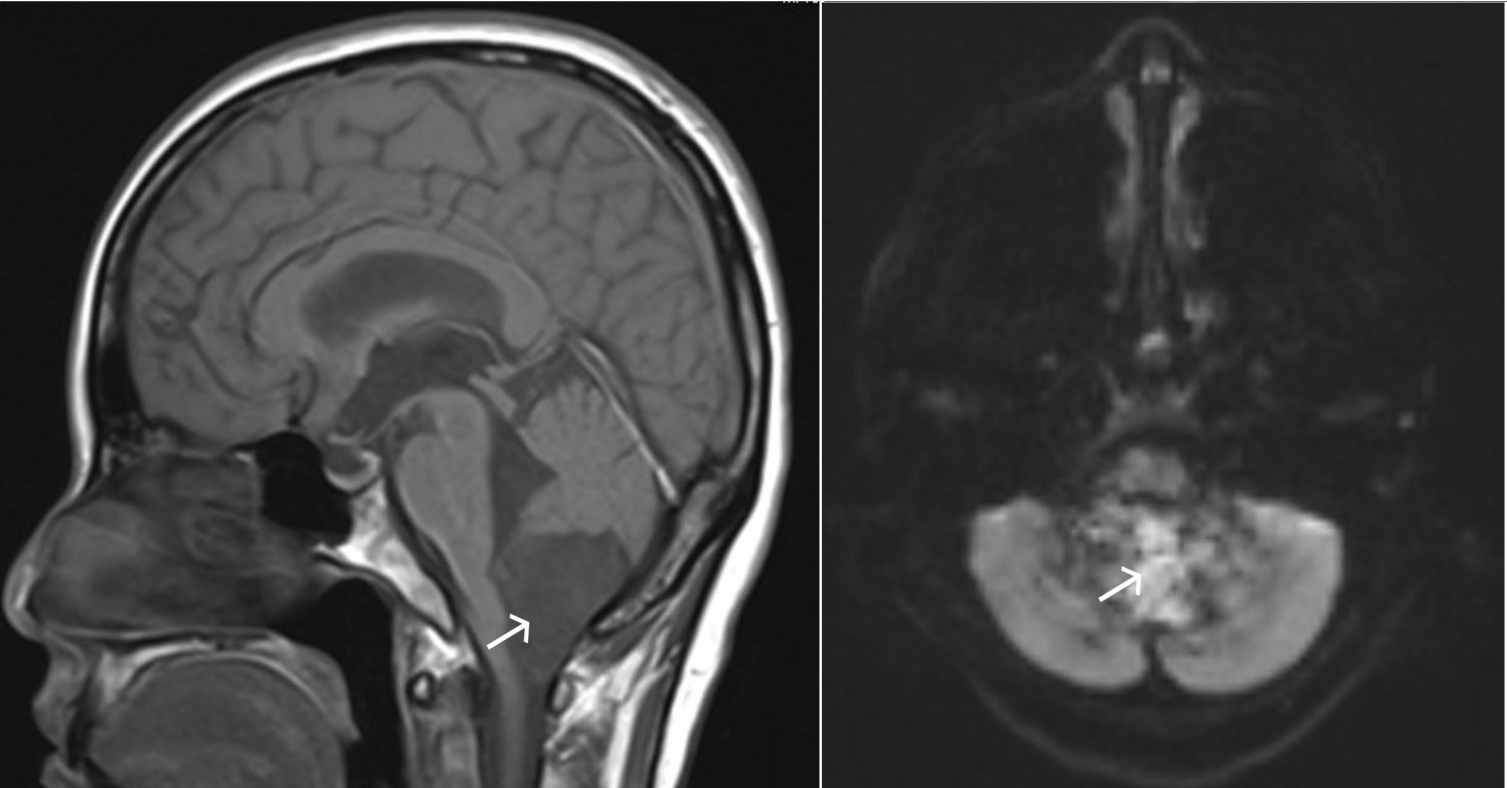
Sagittal T1, axial T2W, and axial DWI MRI of the posterior fossa and cervical spine show a cyst at the craniovertebral junction (white arrow).

A brain MRI scan showed a large lesion in the craniovertebral junction (Figure 1). This lesion was a multicystic tumor. This cyst compressed the medulla and the initial section of the cervical spinal cord. The edema or hydrocephalus was not detected.

### 3.2 Diagnosis and surgical procedures

**FIGURE 2:**
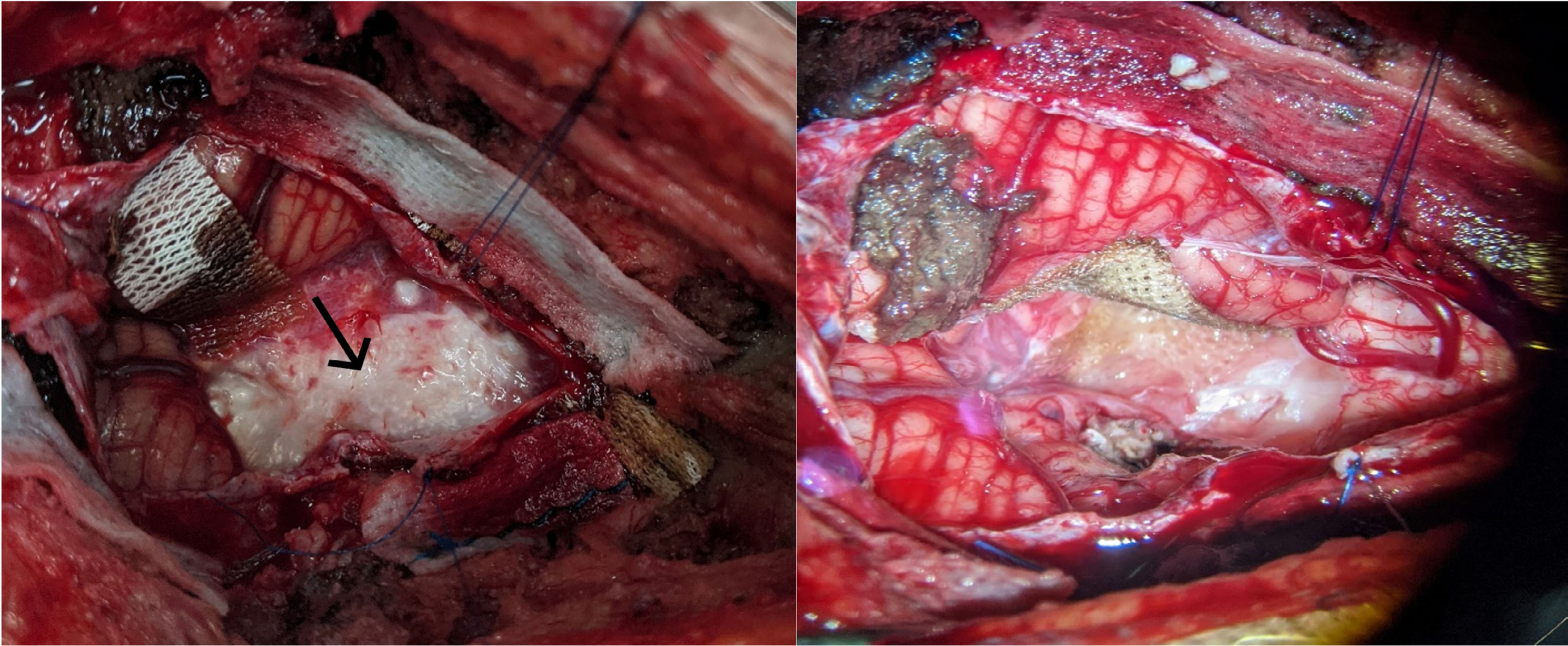
A epidermoid cyst at the craniovertebral junction (left—black arrow) and after being removed (right).

Based on the clinical presentation and MRI finding, the final diagnosis was epidermoid cyst at the craniovertebral junction. We decided to remove the tumor by microsurgery technique. With the prone position, the head was fixed by Mayfield. Because of the lesion involving the upper cervical spine, a suboccipital craniotomy with C1 laminectomy was chosen. Under the microscope, the tumor was resected entirely (Figure 2).

**FIGURE 3:**
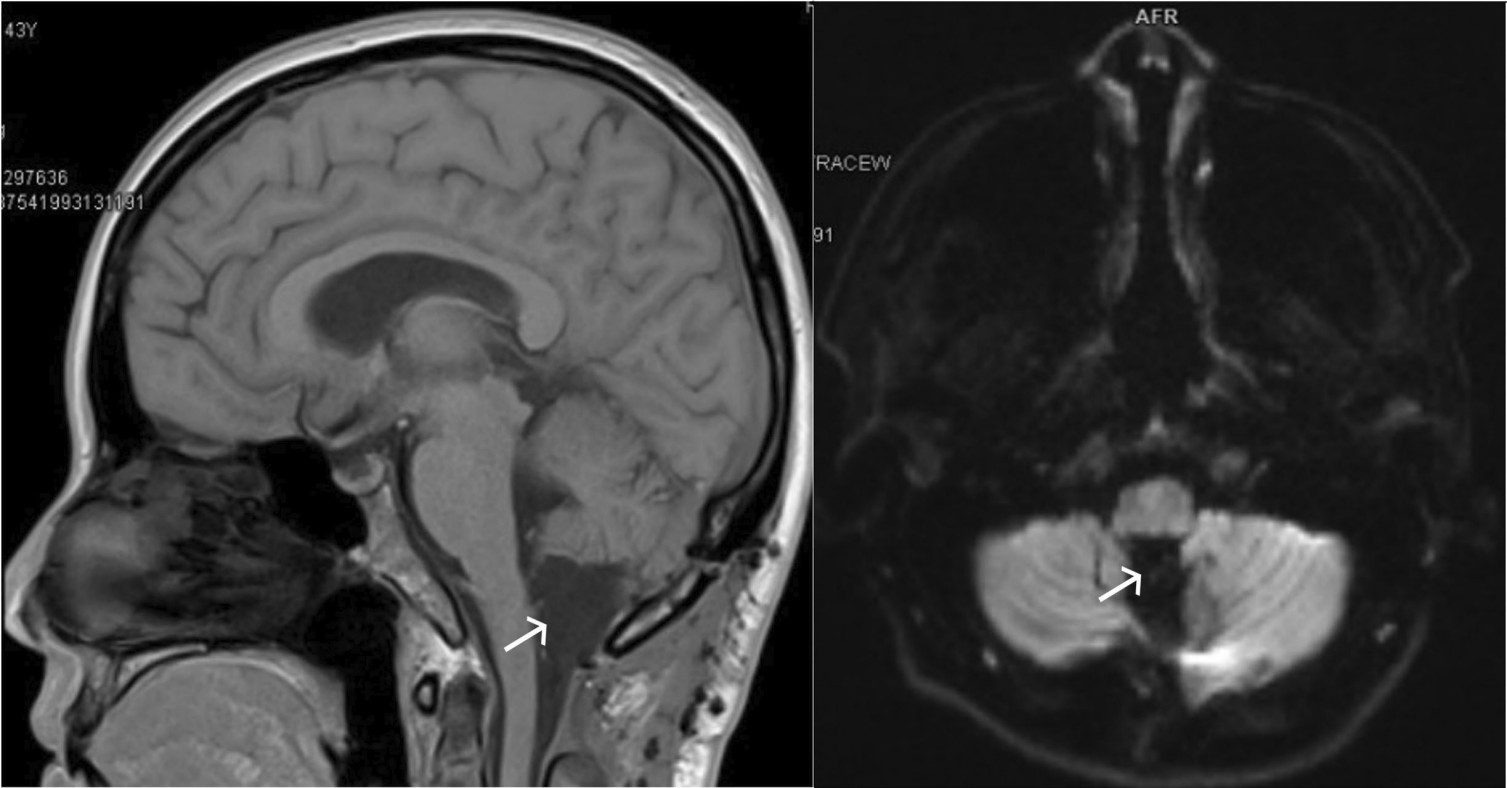
MRI of posterior fossa and cervical spine 1‐month post‐operation confirm no features of a cyst at the craniovertebral junction (white arrow).

The pathology result confirmed the epidermoid cyst. No complication post‐operative was reported. We confirmed no recurrence by MRI of the posterior fossa and cervical spine 1 month post‐operative (Figure 3).

## 4 CONCLUSION AND RESULTS

After the surgery, the patient came back to her normal activities without any neurological deficits. She did not get any complications, including headache, fever, and cerebrospinal fluid leak.

## 5 DISCUSSION

Epidermoid cyst/tumor is a lesion that spreads from the basal surface of the brain to a variety of locations, with the most common being the cerebellopontine angle (40%–50%), suprasellar regions (10%–15%), fourth ventricle (17%), interhemispheric (<5%), and spinal (rare). Epidermoid tumors may be congenital (most common, arising from ectodermal inclusion during neural tube closure) or acquired (post‐surgical or post‐traumatic implantation). According to our knowledge, the epidermoid cyst of the craniovertebral junction is not frequent in the literature. In the series of 234 cases from Vernon et al.,^1^ no case of the craniovertebral junction was reported.

CT shows an epidermoid cyst as isodense lesions concerning the cerebrospinal fluid. It appears hypoattenuating with possible marginal calcification.^4^ Calcification is seen in a minority of cases (10%–25%), and rarely, an epidermoid cyst may be hyperdense due to bleeding, saponification, or high protein content. The interhemispheric epidermoids are reported to have peripheral calcification. They do not enhance and only rarely demonstrate any wall enhancement.

In MRI, this lesion has heterogenic hypointense on a T1‐weighted image, which does not undergo contrast intensification and is usually a hyperintense signal on a T2‐weighted. Still, it is slightly brighter than cerebrospinal fluid on T1‐ and T2‐weighted images.^3^, ^4^ A diffusion‐weighted imaging sequence in which the epidermoid cyst is hyperintense can help differentiate the cyst from the cerebrospinal fluid and non‐specific central nervous system lesions.

The most important thing is to differentiate between epidermoid and arachnoid cysts. On the FLAIR sequence, epidermoid cysts can be recognized because the former show mixed iso‐ to hypersignal intensities but with poor differentiation. DWI offers a finding specific for an extra‐axial lesion by showing a very high signal.^3^, ^4^

In minimal cases, unusual patterns of the epidermoid cyst may be observed on MRI. Such circumstances include white epidermoids, which have a rich protein content and appear with reversed signal intensities with homogeneous high signal intensity on T1‐weighted images and low signal intensity on T2‐weighted images.^3^

The target in treating epidermoid cysts is complete surgical resection.^1^, ^5^ The approach depends on the location of the cyst. In our case, the cyst extends from the posterior fossa to the upper cervical spine. With microsurgery techniques, total resection can be achieved. The extent of removal remains controversial. Although surgery aims for complete removal, few authors advocate total tumor removal. It has been suggested that with microscopic, meticulous, sharp dissection, every bit of the capsule should be removed to prevent a recurrence. However, adherence of the capsule to the important neurovascular structures in and around the tumor often leads to incomplete removal. Coagulation of the residual capsule might help to prevent a recurrence, but it is dangerous if the capsule is near the exquisitely sensitive cranial nerves, brain stem, spinal cord, or vertebral artery at the upper spinal canal.^1^, ^6^

Aseptic meningitis is one of the complications of surgery, the epidermoid cyst. This complication can be prevented by avoiding spillage of contents in the subarachnoid space, complete removal of the tumor wall, and perioperative steroid administration. Communicating hydrocephalus can develop due to meningitis and might require cerebrospinal fluid diversion procedures.

Epidermoid cyst of the craniovertebral junction is rare compared to this lesion's other locations. Based on the characteristics of the epidermoid cyst at MRI, it is not difficult to diagnose this lesion. Microsurgery is the most effective in the complete removal of this tumor. It should be remembered that a conservative subtotal tumor excision is associated with a higher recurrence rate. Preserving adjacent neuronal and vascular structures is always essential for the patient with the expectation of everyday life.

## AUTHOR CONTRIBUTIONS

**Duong Trung Kien:** Conceptualization; data curation; formal analysis; writing – original draft. **Nguyen Manh Hung:** Writing – review and editing. **Nguyen Viet Duc:** Writing – review and editing. **Duong Dinh Tuan:** Writing – review and editing. **Luong Minh Quang:** Writing – review and editing. **Pham Van Duong:** Writing – review and editing. **Tran Viet Hoang:** Writing – review and editing. **Vu Ngoc Anh:** Writing – review and editing.

## FUNDING INFORMATION

The authors did not receive support from any organization for the submitted work.

## CONFLICT OF INTEREST STATEMENT

No potential conflict of interest was reported by the authors.

## CONSENT

Written informed consent was obtained from the patient to publish this report in accordance with the journal's patient consent policy.

## Data Availability

The datasets used and/or analyzed during the current study are available from the corresponding author on reasonable request.
